# The effect of serum origin on cytokines induced killer cell expansion and function

**DOI:** 10.1186/s12865-023-00562-3

**Published:** 2023-09-01

**Authors:** Zahra Jabbarpour, Seyed Sajjad Aghayan, Kobra Moradzadeh, Sasan Ghaffari, Naser Ahmadbeigi

**Affiliations:** 1 Gene Therapy Research Center, Digestive Disease Research Institute, Tehran University of Medical Sciences, Shariati Hospital, North Kargar Ave, Tehran, 14117 Iran; 2 Department of Hematology, School of Allied Medical Sciences, Tehran University of Medical Sciences, Tehran, Iran

**Keywords:** Cytokine-induced killer cells (CIK), Cell expansion, Good Manufacturing Practice (GMP), Cell therapy

## Abstract

**Background:**

Cytokine-induced killer (CIK) cells have shown promising results in adoptive immunotherapy. However, serum may play a determining role in the large-scale expansion of these cells for clinical applications. According to Good Manufacturing Practice (GMP) guidelines to reduce the use of animal products in cell-based therapies; therefore, this study sought to investigate the impact of serum origin and the reduced serum concentration on the pattern of cell expansion and function.

**Methods:**

Peripheral blood mononuclear cells (PBMCs) isolated from a healthy donor were expanded based on the CIK cell expansion protocol. The cell culture medium was supplemented with three types of sera comprising fetal bovine serum (FBS), human serum (HS), or human-derived platelet lysate (hPL) at different concentrations (10%, 5%, and 2.5%). The proliferation kinetics for each group were investigated for 30 days of cell culture.

**Results:**

Cell proliferation in 10% concentration of all sera (hPL, FBS, HS) was higher than their lower concentrations. Moreover, hPL was significantly associated with higher expansion rates than FBS and HS in all three concentrations. Furthermore, cells cultured in hPL showed higher viability, cytotoxicity effect, and CIK CD markers expression.

**Conclusion:**

hPL at a concentration of 10% showed the best effect on CIK cell proliferation and function.

**Supplementary Information:**

The online version contains supplementary material available at 10.1186/s12865-023-00562-3.

## Background

Adoptive immunotherapy has grown dramatically as an effective therapeutic modality for cancer treatment in recent decades. Generally, immunotherapy approaches stimulate the immune system by increasing the activity of any components [1]. Immunotherapy strategies can be classified into several categories: cellular immunotherapy (Dendritic cells, T cells, Natural killer cells, Cytokine-induced killer cells (CIK)), monoclonal antibodies, cytokine therapy, DNA vaccines, viruses, and bacteria-mediated immunotherapy. The most immune cells used in adoptive cell therapies are DC and CIK cells [2].

CIK cells are a heterogeneous T-cell population with a higher efficacy profile in cancer immunotherapy [3]. It has been argued that CIK cells can eliminate tumor cells in a non-MHC-restricted manner [4, 5]. This cellular product consists of three subsets of cells, including CD3 + CD56+, CD3 + CD56- and CD3-CD56 + cells. The CD3 + CD56 + subset has been considered the CIK effector cells [6].

In clinical settings, expansion of CIK cells must be performed according to good manufacturing practice (GMP) guidelines. Therefore, one of the most crucial principles has been instructed to use animal-origin-free materials culture conditions and cryopreservation procedures [5, 7, 8]. One of the most important constituents of cell culture condition is serum, a source of essential growth factors. Fetal bovine serum (FBS) has been a widely used serum supplement for cell culture. However, since the use of animal derivatives can lead to adverse events, the GMP guidelines generally recommend abandoning FBS use [9, 10].

Hence, for cell therapy in clinical settings, human-originated serum sources, such as human serum (HS) and human platelet lysate (hPL), can be considered proper FBS alternatives. Furthermore, studies have shown that hPL contains various growth factors and cytokines with vigorous mitogenic activity, making it an ideal supplement for cell culture [11–13]. To achieve the greatest anti-cancer effects, CIK cells should be administered in large numbers (about 5 × 10^10^ per course) to the patients. Therefore, the expansion of this number of cells requires more than 10 L, large quantities of culture medium, and serum [14].

**Fig. 1:**
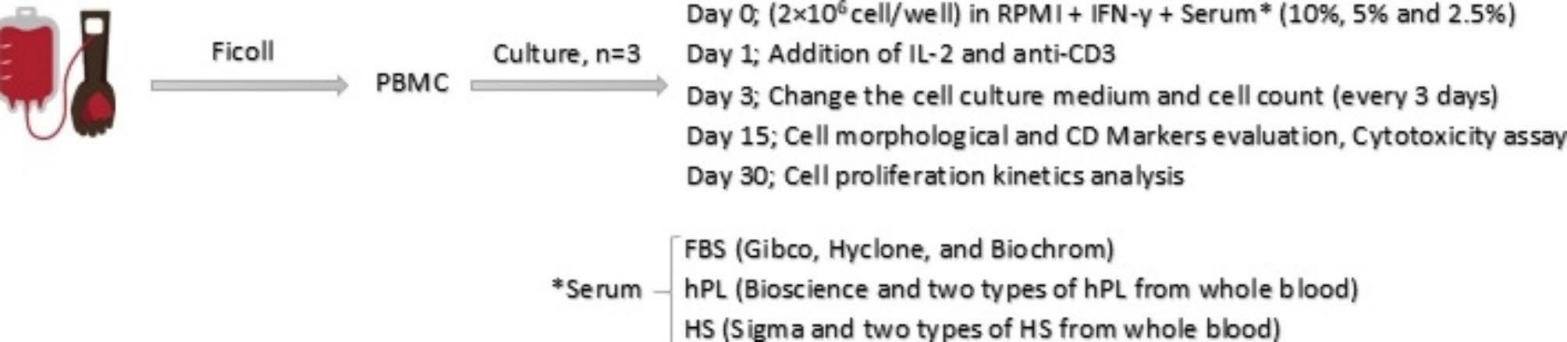
Methodological diagram. Graphical representation of the experimental steps. The effect of three different sera (FBS, hPL, and HS) on the expansion of CIK cells

For clinical use, we need to find a GMP-grade substitute for FBS to expand these cells in large numbers. Hence, this study was designed to compare the impact of human-originated serums with FBS and delineate the effect of different serum concentrations on the expansion and function of CIK cells, Fig. 1.

## Methods

The Tehran University of medical sciences ethics committee has approved this study for ethics in biomedical research (IR.TUMS.VCR.REC.1397.882) in accordance with the Declaration of Helsinki. Informed consent was obtained from all individual participants included in this study.

### PBMC isolation

First, 10 ml of peripheral blood was collected and transferred into a sterile tube containing 40 U/ml heparin (Aburaihan Pharmaceutical Co, Iran). Next, peripheral blood mononuclear cells (PBMCs) were isolated from the blood using a density gradient separation medium (1.077 g/ml, Ficoll-Hypaque, GE Healthcare, 17-1440-02) via centrifugation at 400 g for 30 min. The cells (buffy coat) were then washed and centrifuged three times to remove platelets (300 g / 5 min, 200 g / 10 min, and 180 g / 10 min at room temperature, respectively).

### Serum preparation

The sera used in this study were purchased from Gibco (10270-106), Hyclone (SH30070.03), and Biochrom (S 0115) for FBS, Bioscience (PLS-100.02b) for hPL, and Sigma (S1-M 637,810) for HS. In addition to these commercial sources, we also prepared human sera in our lab according to the following standard protocols;

For HS preparation, after collecting the whole blood from two donors (autologous and heterologous), the blood samples were placed on a bench at room temperature to clot for 30 min. Next, the clotted blood was centrifuged at 1000–2000 g for 15 min. After centrifugation, the supernatant (serum) was carefully aspirated and transferred into a clean tube. Finally, the serum sample was aliquoted and stored at -80 °C. Written informed consent was obtained from two donors.

For hPL preparation, platelet concentrates were taken from platelet rich plasma (PRP) units frozen at − 80 °C and then thawed in a water bath at 37 °C. This step was repeated three times. The lysate was cleared via centrifugation (2200 g, room temperature, 20 min) to remove membrane fragments, and the supernatant was filtered using 0.45 μm and 0.2 μm filters, respectively. The serum sample was aliquoted and stored at -20 °C. Before adding the serum to the culture medium, 2 U/ml heparin as an anticoagulant was added to prevent hPL gel formation. Written informed consent was obtained from two donors.

### CIK cell culture

Isolated PBMCs were transferred into a flask containing RPMI medium (Bioidea, BI-1006-05). After incubation at 37 ^o^C in a 5% CO2 atmosphere for 2–4 h, the non-adherent cells were then cultured in 24-well plate (at the density of 2 × 10^6^ cell/well containing RPMI medium supplemented with 100 IU/ml penicillin/streptomycin (Bioidea, BI-1203), 1000 IU/ml IFN-γ (Miltenyi Biotech, 130-096-482), and 10%, 5%, and 2.5% of one of the following sera: FBS, hPL and HS on day 0. Hence, this study compares the cell expansion kinetics of 9 groups based on the mentioned serum concentrations and serum source (Fig. 1).

On the following day (day 1), 300 IU/ml of rh-IL-2 (Thermo Fisher Scientific, 130-097-748) and 50 ng/ml of functional-grade anti-CD3 antibody (Miltenyi Biotech, 130-093-387) were added to the medium of all wells. Thenceforward, every 2–3 days, the wells were replenished with a fresh medium containing 300 U/ml IL-2. After reaching the confluence, the cells were subcultured into new wells with the same seeding density. The cell culture continued up to 30 days. Every day, cell density and morphological characteristics were inspected under an inverted microscope.

### Cell counting and viability evaluation

During the cell expansion, cells were counted every three days (as a time point; TP). As described before [15], Fold expansion, population doubling (PD), cumulative population doubling (CPD), and cell viability were assessed for each well to evaluate CIK cell proliferation. Fold expansion was calculated by dividing the cumulative number of cells expanded in a well on the initially seeded number of PBMCs. PD was calculated based on the following equation: PD = 3.32 (log N1 - log N0), in which N1 represents the number of cells counted at each time point, and N0 represents the number of initially seeded cells. CPD was calculated by adding the PD of each time point to the PD of previous time points. All the experiments were performed in triplicates for each group (as described above).

### Flow cytometry analysis

The expression of CIK CD markers on the CIK cells was evaluated by flow cytometry on the 15th day. For this purpose, 2 × 10^5^ CIK cells were washed once with PBS, and then were suspended in a tube containing PBS containing 3% bovine serum albumin (Sigma, A7284-10ML). Immediately, each tube was incubated at 4^o^C with anti-human CD3-PerCP (Miltenyi Biotec, 130-094-965) or anti-human CD56-PE (BD, BD 561,903), and appropriate isotype controls [Mouse IgG2a isotype PerCP (Miltenyi Biotec, 130-099-190) and mouse IgG1 isotype PE (BD, BD 556,650)]. Next, the mixture was incubated at 4 °C and washed twice with PBS. In addition, the cell pellet was washed twice and then were suspended in PBS. The cell phenotype was then analyzed using BD FACSCalibur flow cytometer (BD Biosciences, USA), and the resultant flow cytometry data were analyzed using. Flowjo 8 software (FlowJo LLC, USA) was used to analyze flow cytometry data.

### Cytotoxicity assays

According to the CIK cell expansion results in hPL, the cytotoxic effects of CIK cells cultured in hPL at 10% and 5% concentrations were investigated on target cells. The CIK cells used for the assay were on the 15th day of expansion. Two hematological cell lines (K562 and Raji) were selected as target cells. Target cells were labeled using Carboxyfluorescein succinimidyl ester (CFSE) dye (Invitrogen, C34554) before co-culturing with CIK cells. To this end, 1 × 10^6^ target cells were obtained from cell culture for CFSE staining. After washing with PBS, the cells were resuspended in 5% FBS-PBS solution. CFSE dye was added to the cell suspension to give a final concentration of 5 μg/ml.

The cell suspension was then incubated at 37 °C in a bain-marie for 10 min. Next, neutralization was performed by the addition of RPMI 1640 medium supplemented with 10% FBS. After washing, the cells (5 × 10^4^) were co-cultured with CIK cells in 48 well-plate at three effectors to target (E:T) ratios of 40:1, 20:1, and 10:1 overnight in RPMI with 10% FBS and incubated at 37 °C in 5% CO2. After 24 h, cells were harvested for propidium iodide (PI) (BioLegend, 640,906) staining. After the PBS washing, cells were incubated with PI-working solution (50 μg/mL PI, RNase A, sodium citrate, and Triton X‐100) at 4 °C for 30 min in the dark. Finally, the percentage of cells labeled with PI in the target cell population was assessed by flow cytometry.

### Statistical analysis

All experiments were performed in triplicates. Data were analyzed using GraphPad Prism version 8. The results of each variable were reported as the mean ± SEM. Data were analyzed using Two-Way Repeated Measures ANOVA and Two-Way ANOVA followed by Tukey test. A p-value less than 0.05 was considered statistically significant.

## Results

### Morphological characteristics of PBMCs

**Fig. 2:**
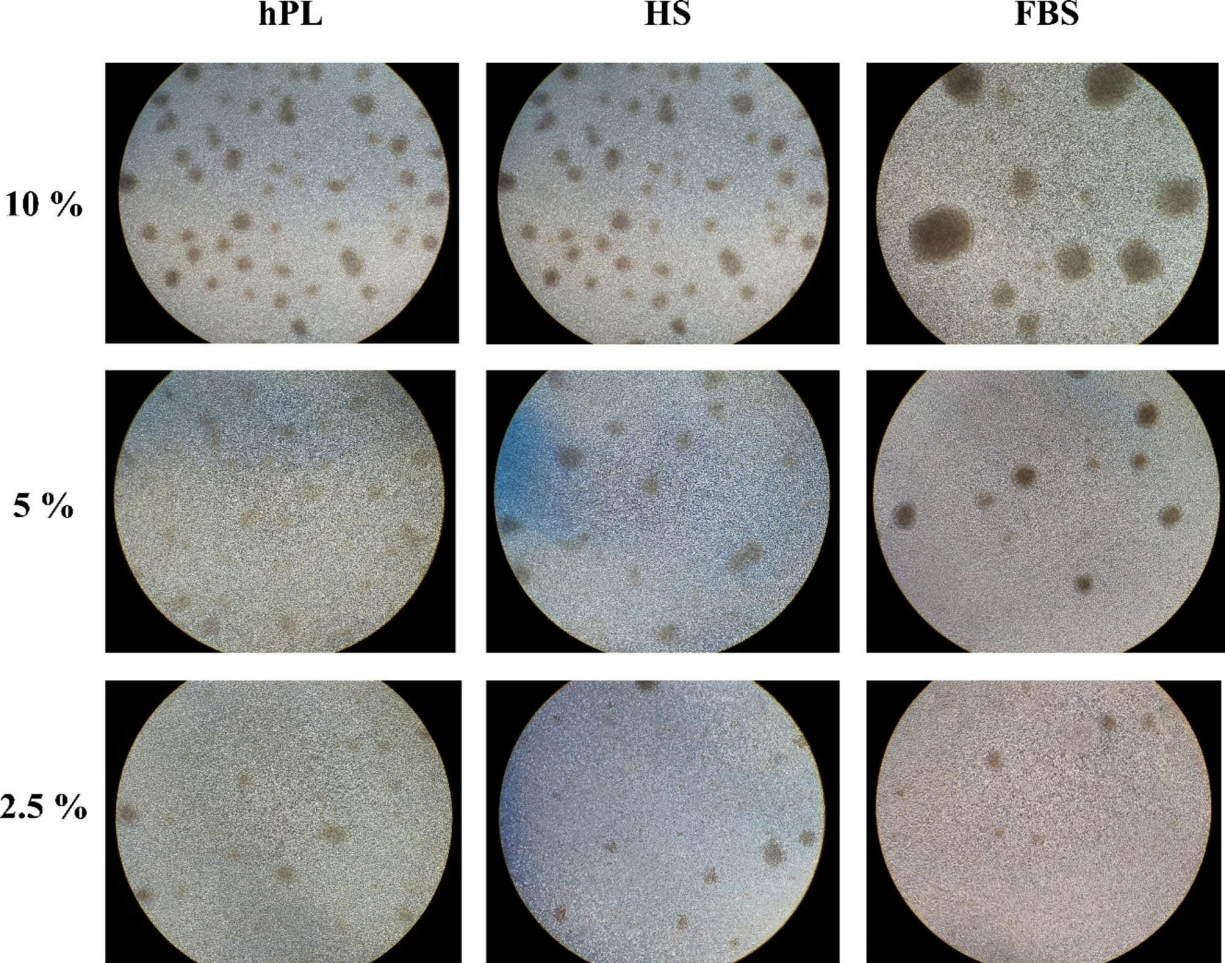
The effect of three different sera on the morphological characteristics of CIKs’ aggregates on the 15th day. In the hPL group, a large number of small sizes, circular, and light-colored aggregates were observed. The analysis was performed at an inverted microscope with 10X magnification

PBMCs were isolated and counted from a donor. The 10 ml collected blood provided roughly 1.5 × 10^6^ mononuclear cell/ml of the extracted blood with more than 98% viability. Our microscopic observations showed that cells have a tendency to form aggregates. The aggregates were visible during the entire culture period, especially during the first two weeks. Some differences were observed in the morphology of the cells between the groups. In the FBS group, a few aggregates with large size, dense, and irregular edges were seen, whereas, in the hPL and HS, a large number of small sizes and circular aggregates were observed. Interestingly, the number of aggregates decreased at lower concentrations of all three types of sera (Fig. 2).

### CIK cells kinetics

Data analysis at the last TP (TP10) showed fold expansion, and CPD was significantly higher in the 10% hPL group compared to the FBS group (p < 0.0001). The 10% hPL group also demonstrated higher fold expansion (p < 0.0001) and CPD (p < 0.05) than the 10% HS group. Additionally, despite the increase in fold expansion and CPD in the 10% HS group compared to the FBS group, no significant difference was observed between the two groups. In PD/Day, no difference was observed between the three groups and all three groups at a concentration of 10% showed a maximum expansion at TP 3.

**Fig. 3:**
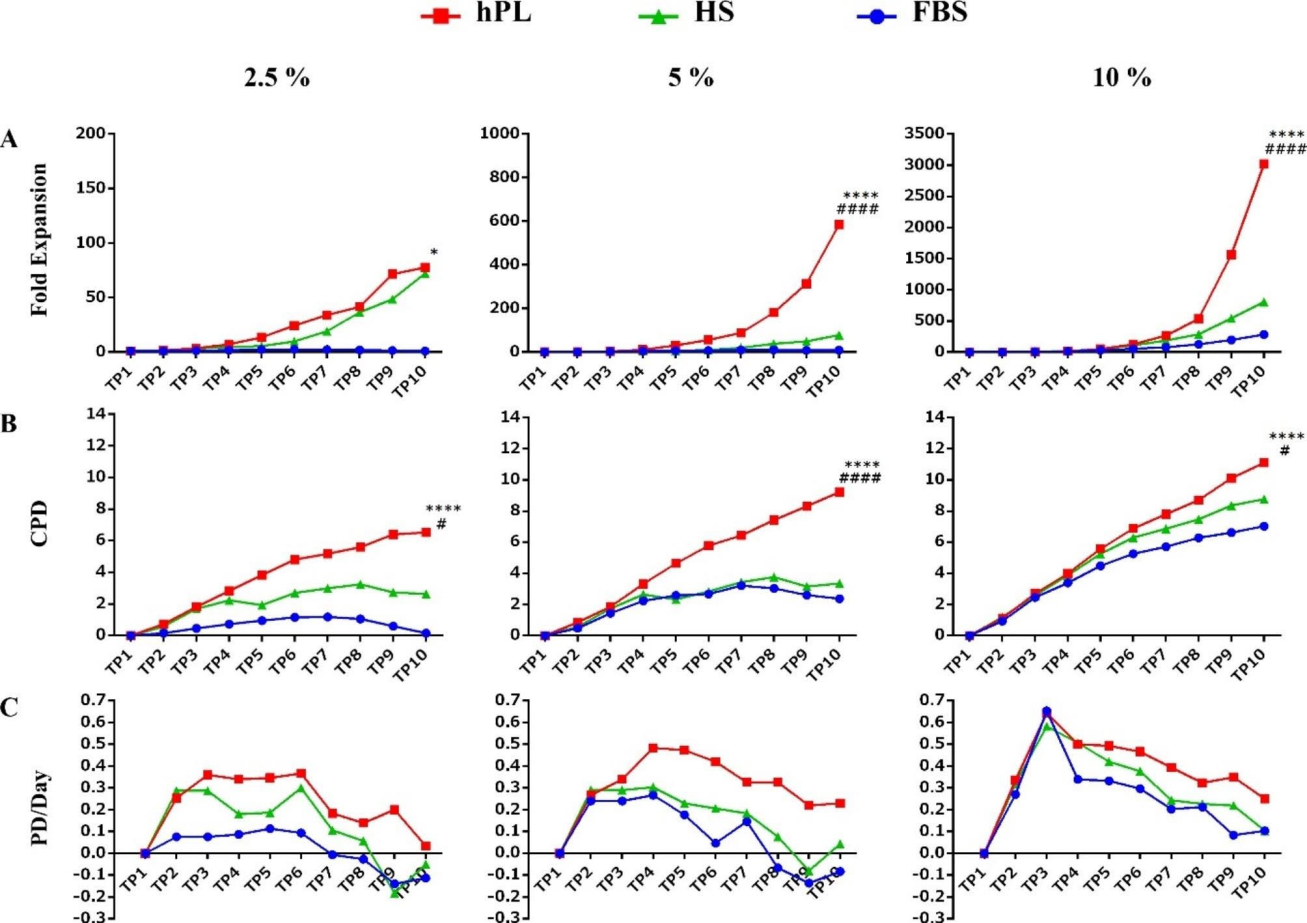
The effect of three different sera on the kinetics of proliferation of CIK cells. (**A**) The maximum expansion rate was seen in 10% hPL. (**B**) Low serum concentration dramatically reduced cell proliferation in the FBS group. (**C**) The maximum cell expansion rate occurred in all three groups at a concentration of 10% during the ninth and tenth days. Data analysis indicates that fold expansion and CPD of cultured cells in a medium containing hPL at three concentrations are significantly more than the FBS group at the end of culture. At the same time, there is no significant difference between FBS and HS groups. Also, in the PD/Day, maximum expansion was seen in time point 3 (TP3) in all groups at a concentration of 10%. The presented results are related to the differences between groups in TP10. (n = 3); These data were analyzed using Two-Way Repeated Measures ANOVA followed by Tukey test and shown as mean ± SEM. * as hPL compared with FBS group, # as hPL compared with HS group, and $ as HS compared with FBS group. *p < 0.05, **p < 0.01, ***p < 0.001, ****p < 0.0001

**Fig. 4:**
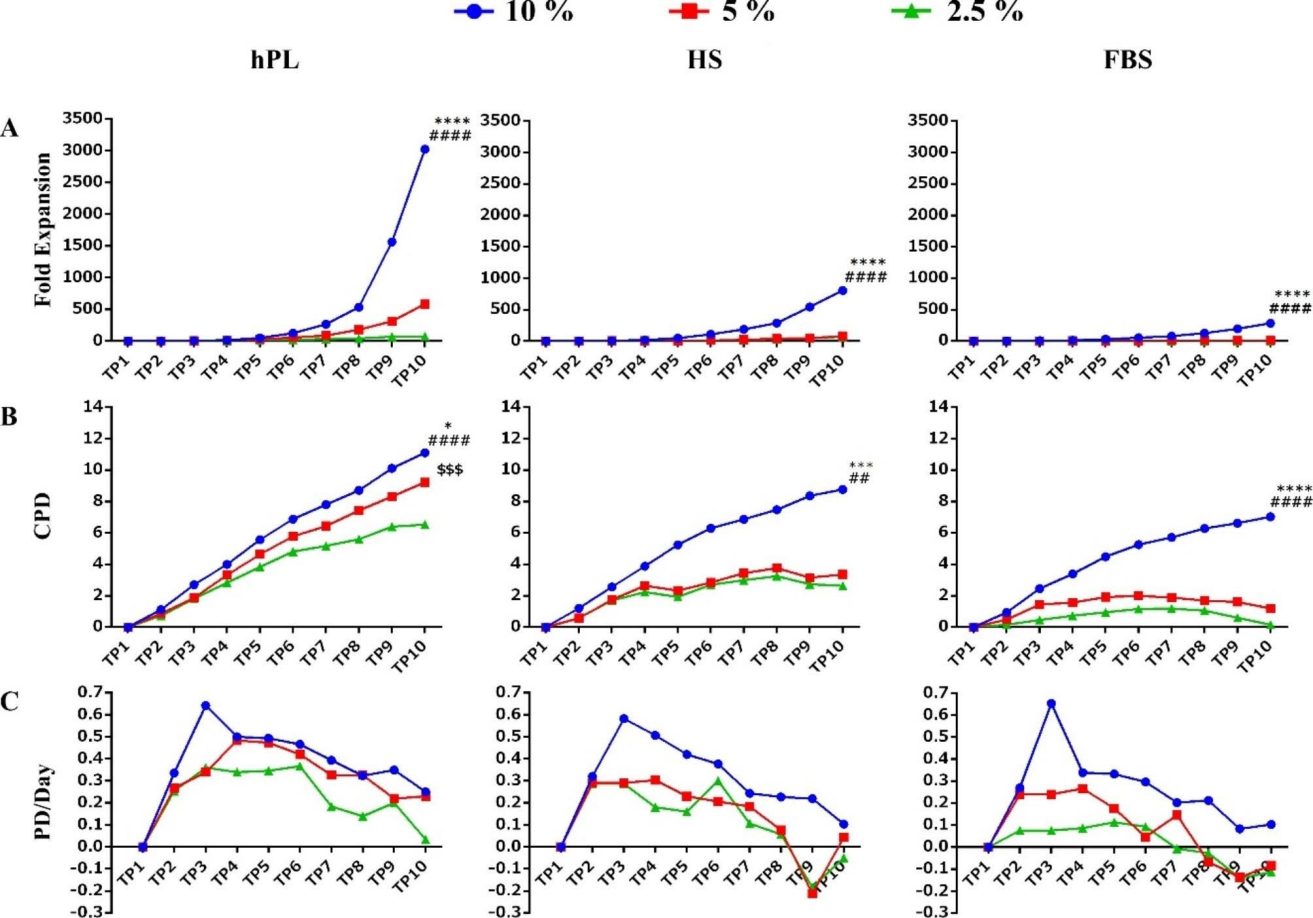
The effect of low concentrations of sera on CIK cell growth kinetics in three study groups. (**A**) The maximum expansion rate was seen in a 10% concentration of sera. (**B**) hPL in lower concentration (5%) did not affect cell expansion. (**C**) The maximum cell expansion rate occurred in all three groups during the ninth and tenth days. The fold expansion and CPD in cultured cells in a medium containing 10% hPL are significantly more than 5% and 2.5% concentrations. These results were the same as HS and FBS since the 10% concentrations proved to be more efficient in CIK cell expansion than lower concentrations. (n = 3); These data were analyzed using Two-Way Repeated Measures ANOVA followed by Tukey test and shown as mean ± SEM. * as 10% compared with 5% group, # as 10% compared with 2.5% group and $ as 5% compared with 2.5% group. *p < 0.05, **p < 0.01, ***p < 0.001, ****p < 0.0001

Fold expansion and CPD of CIK cells were significantly higher when cultured at 5% and 2.5% of hPL than when cultured in the same concentration of FBS or HS groups except for fold expansion of 2.5% hPL 2.5% HS. In addition, there was no significant difference in PD/Day between all three groups (FBS, hPL, and HS) at concentrations of 5% and 2.5% (Fig. 3). However, in all three types of sera, fold expansion and CPD showed a significant increase at a concentration of 10% compared to lower concentrations (5% and 2.5%) (Fig. 4).

### CIK viability and identity evaluation

**Fig. 5:**
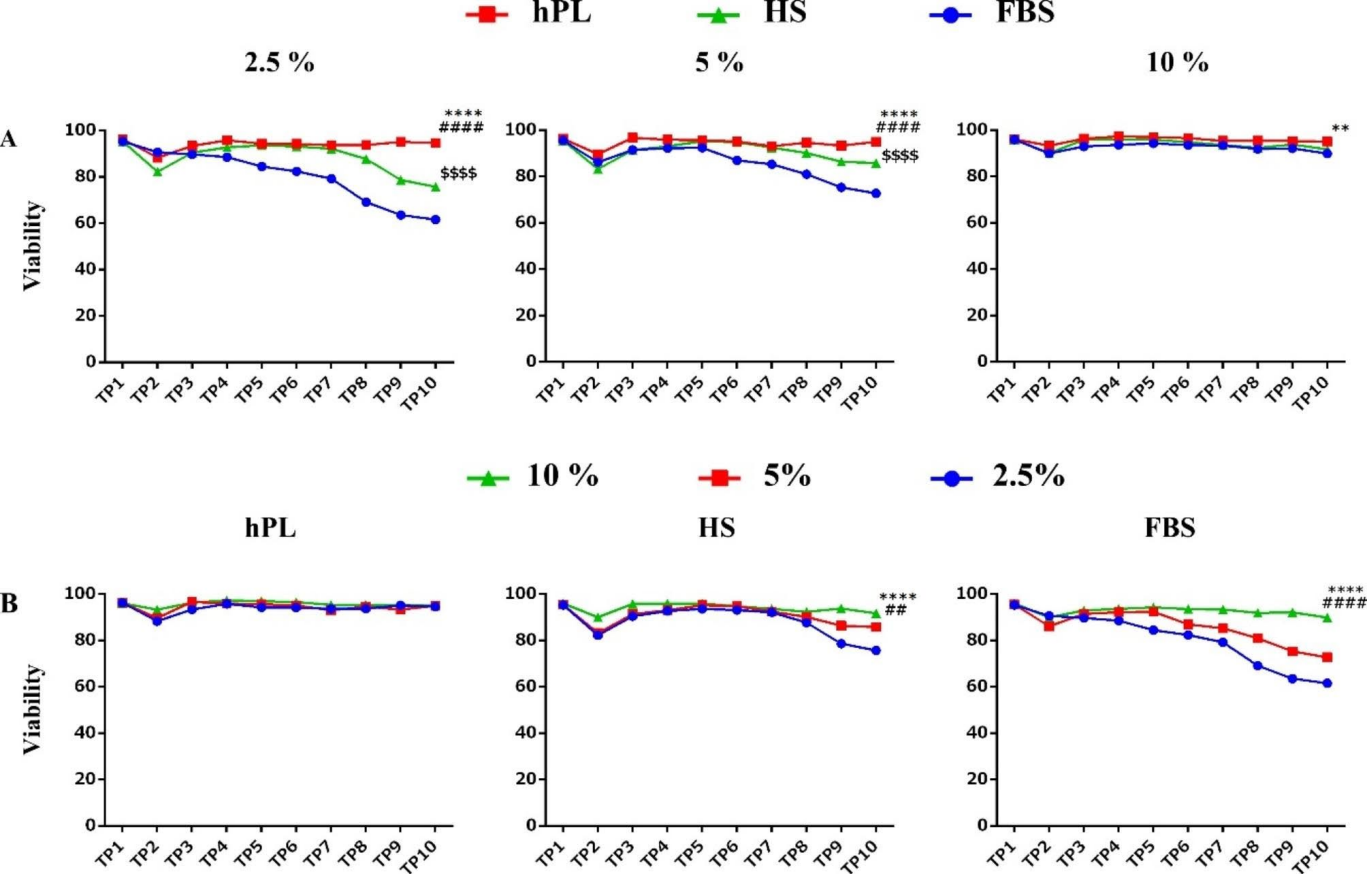
Viability of CIK cells in studied groups. (**A**) Cell viability at a concentration of 10% in all three sera is more than 90%. (**B**) Low serum concentration reduced cell viability in the FBS group. The data analysis showed that the viability percentage of cells cultured at different concentrations of hPL was significantly higher than the other two sera. In all three concentrations of hPL, CIK cells showed significantly higher viability than the other two groups (HS and FBS). Besides, HS in concentrations 5% and 2.5% exhibited significantly higher viability than FBS. No significant difference in viability was observed in different concentrations of hPL. The presented results are related to the differences between groups in TP10. (n = 3); These data were analyzed using Two-Way Repeated Measures ANOVA followed by Tukey test and shown as mean ± SEM. * as hPL compared with FBS group, # as hPL compared with HS group, and $ as HS compared with FBS group. *p < 0.05, **p < 0.01, ***p < 0.001, ****p < 0.0001

The results showed that the viability of cells cultured with hPL was more than 90% in all three concentrations (10%, 5%, and 2.5%) at all-time points and was significantly higher than the viability of cells cultured the other two sera. It is noteworthy that at 5% and 2.5% concentrations, the viability of cells cultured in a HS-supplemented medium was significantly higher than those in FBS (Fig. 5).

**Fig. 6:**
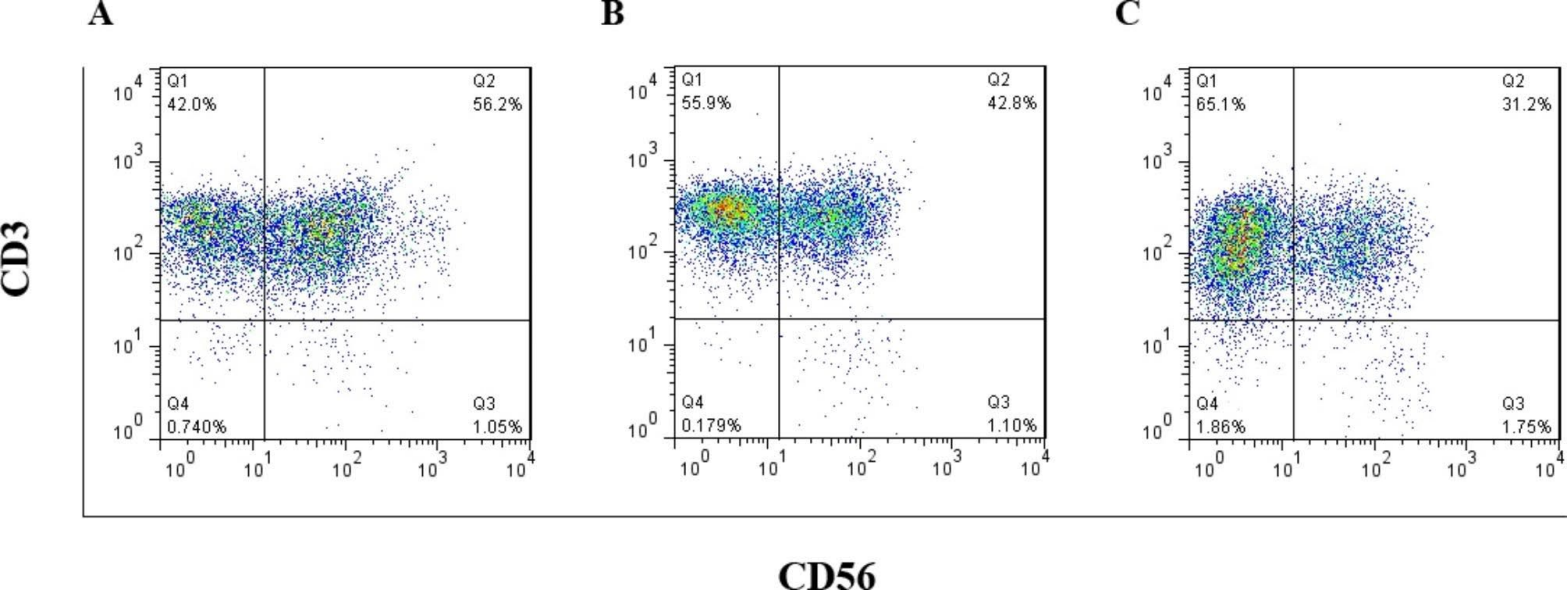
CD markers of CIK cells. The surface expression of CIK CD markers (CD3/CD56) after isolation and culture in (**A**) hPL 10%, (**B**) hPL 5%, and (**C**) hPL 2.5% were evaluated by flow cytometry

To investigate CIK cells’ nature, we examined their CD markers’ expression, including CD3+, CD56+, and CD3 + CD56 + markers using flow cytometry. Expression of these markers was decreased at 5% and 2.5% concentrations of serum (Table S1). However, in the 5% hPL group, more than 40% of the cells express CIK CD markers (Fig. 6).

### CIK cells cytotoxicity

**Fig. 7:**
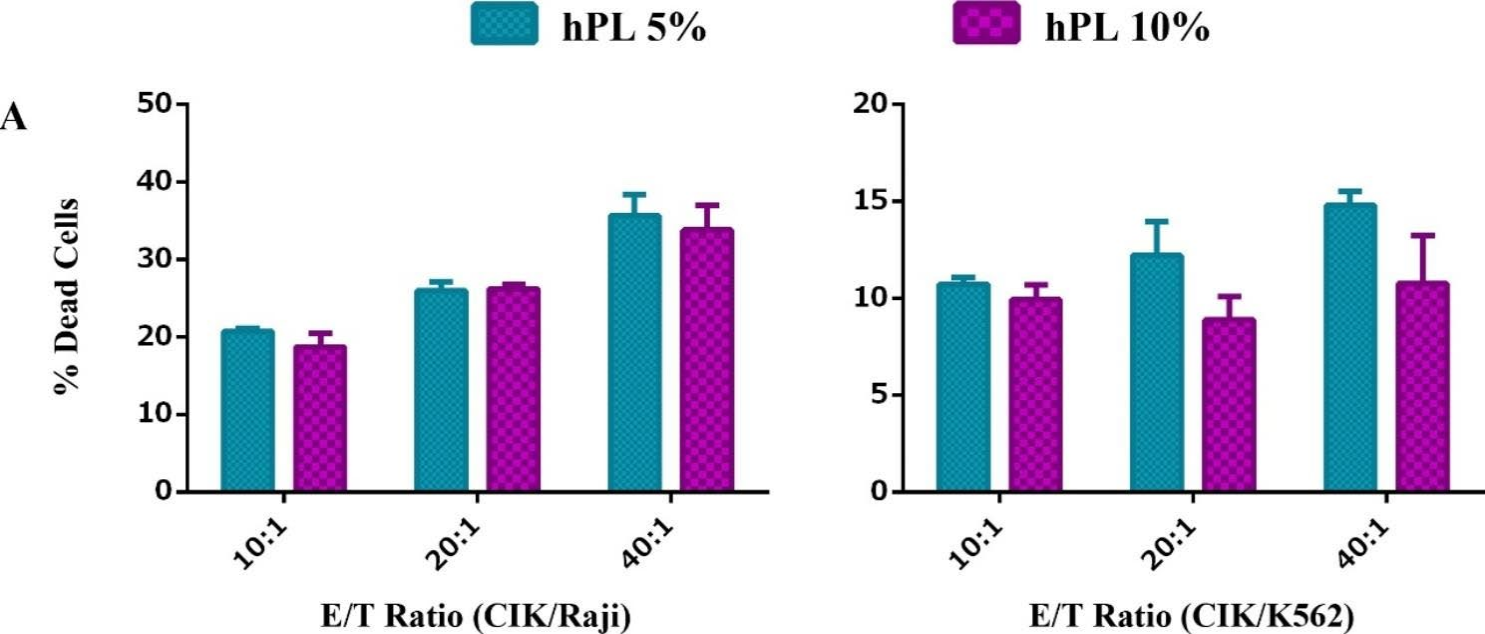
The effects of low concentrations of sera on CIK cell cytotoxicity on the 15th day. These data were analyzed using Two-Way ANOVA followed by Tukey test and shown as mean ± SEM. (n = 3); There was no significant difference in the percentage of dead cells in different effector to target ratios in all two target cells. hPL in lower concentration (5%) did not have any adverse effect on CIK cell cytotoxicity

The cytotoxicity effect of CIK cells cultured in 10% and 5% hPL on the two cell lines (K562 and Raji) was examined. Based on our results, no significant difference was observed between the cytotoxic effect of cultured CIK cells in 10% and 5% of hPL on all two cell lines in different effector ratios to target (Fig. 7).

## Discussion

Immunotherapy has recently emerged as a promising method in the treatment of malignancies. In this regard, CIK cells are used as one of the ideal options for adoptive immunotherapy [16]. These cells were obtained from the ex vivo expansion of PBMCs. Expanded CIK cells are a heterogeneous population of CD3^+^CD56^+^, CD3^+^CD56^‑^ and CD3^‑^CD56^+^ lymphocytes. CD3^+^CD56^+^ CIK cells exhibit potent MHC-unrestricted cytotoxicity against solid and hematological malignancies [17].

Living cells as therapeutic products always face limitations such as cell viability and function. On the other hand, cell therapy products must follow the requirements in GMP guidelines [5]. According to the regulatory authorities’ recommendations, animal-derived products should not be used as far as possible in the process of clinical applications of cellular products. FBS is widely used to culture and expansion of different cell types. Disadvantages of using FBS for clinical applications include batch-to-batch variation, disease transmission, and xeno-immunization. Studies show that xeno-free sera, such as hPL or HS, can be used for different cell cultures [18–20].

CIK cells are usually used in large numbers for adoptive cell therapy; hence, the expansion of many cells requires high amounts of medium and serum [14]. Accordingly, reducing the amount of serum used without affecting the viability and function of CIK cells can play an essential role in reducing the cost of large-scale ex vivo expansion of these cells. This study tried to optimize a xeno-free culture protocol for CIK cells. These cells were expanded in a medium containing different sera concentrations separately. Our results indicate that the fold expansion and CPD in 10% of all three sera are significantly more than other concentrations. Furthermore, the fold expansion and CPD in the hPL group are significantly more than the other groups (at all three concentrations).

Considering the feasibility of expanding cells with less than 10% hPL [21], we compared CIK cells culture with different hPL concentrations (5% and 2.5%). Our results suggest that 5% of hPL can be as efficient as 10% hPL for CIK expansion. CPD showed a significant increase in hPL at concentrations of 5% compared to 2.5%, and despite increasing the fold expansion in hPL 5–2.5%, no significant difference was observed between them. In all three concentrations of hPL, CIK cells showed significantly higher viability than the other two groups (HS and FBS), while the viability of cells at three different hPL concentrations did not show a significant difference. Moreover, a few aggregates with large size, dense, and irregular edges were seen in FBS group, whereas, in the hPL and HS, many small sizes and circular aggregates were observed, and the number of aggregates decreased at lower concentrations of all three types of sera. In a study by Saury et al., human muscle stem cells (hMuStem) expanded in 10% FBS, HS, or hPL for 12 days [22]. They reported more CPDs for hMuStem cells cultured in HS than FBS. Cells cultured in hPL also expanded more than FBS, but the difference was not significant. Rebollo et al. demonstrated that expansion of MSCs in 10% hPL significantly increases cell proliferation compared to fetal calf serum (FCS) and dramatically alters cellular morphology [23].

In another study, Dessels et al. expanded adipose-derived stromal cells (ASCs) in four concentrations of hPL (1%, 2.5%, 5%, and 10%) and compared their cell number, size, and morphology to 10% FBS. After seven days, they showed that ASCs supplemented with 10% hPL proliferated significantly more than ASCs supplemented with 10% FBS. Additionally, they demonstrated that ASCs in 10% hPL proliferated the same as when cultured in 5% hPL [24]; these findings are in accordance with our results. When cells are stimulated to proliferate, they experience three growth phases: lag, exponential, and plateau. Considering the different nature of cells, they must expand to the plateau phase for a better conclusion [25, 26].

Hence, we expanded cells for 30 days. The graphs show how maintaining a higher culture period allows for a better portrayal of the results. Other studies expanded ASCs in hPL and FBS. They also revealed that, compared to hPL-cultured ASCs, FBS-cultured cells had a significantly slower population doubling time and lower CPD [27, 28]. Shirzad et al. used umbilical cord blood (UCB)-derived platelet lysate (UC-PL) as a substitute to FBS in 1%, 5%, 10%, 20%, and 30% concentrations [29]. UCB-PL excelled in expanding MSCs compared to FBS. However, it could only be stated regarding 5% and 10% doses of UCB-PL. They stated that lower than 5% and higher than 10% PL concentrations could affect cell expansion. Specially with 30% UCB-PL concentration, excessive cell toxicity was reported.

Mohammadi et al. expanded Wharton’s jelly-derived MSCs in hPL ranging from 1 to 10% and compared them to 10% FBS, demonstrating a superior expansion in 5% and 10% hPL than FBS [30]. Nevertheless, on the contrary, Castiglia et al. showed that human serum was not an appropriate alternative to FBS. It has an undesirable effect on the CIK viability, growth, and identity. They reported that it probably showed such results because of the batch-to-batch variation [31]. CD3^+^CD56^+^ cells are the more effective subset of CIK cells [32, 33]. In this study, flow cytometry analysis of various hPL concentrations showed that CIK cells expanded under 10% hPL express CD3^+^CD56^+^ marker more than 10% concentration of other sera. Our results indicated, not only the expansion potential of 5% hPL is almost on par with 10% hPL, but it also allows for the differentiation of CD56^+^ CIK cells rather than CD56^−^ cells. Besides, the cytotoxic properties of CIK cells at lower concentrations were no different from those at 10% concentration. In other words, hPL could be an appropriate alternative to FBS, even at a lower concentration (5%) did not adversely affect the proliferation, viability, and function of CIK cells.

## Conclusions

For many reasons, using human-based sera such as hPL to nourish cells in vitro is more advantageous than animal-based sera. It is more emphasized when practicing GMP is desired. The fact that hPL is a valuable source of growth factors and can be attained from expired batches of platelets makes it a fascinating choice compared to other serum sources. In conclusion, the present study confirms that the importance of using hPL as an FBS substitute has better results in CIKs proliferation and function. Also, hPL at low concentrations has no adverse effects on the growth and function of these cells. Our data can be considered in the context of cell therapy with the aim of large-scale cell expansion.

### Electronic supplementary material

Below is the link to the electronic supplementary material.

Supplementary Material 1

Supplementary Material 2

Supplementary Material 3

Supplementary Material 4

Supplementary Material 5

Supplementary Material 6

Supplementary Material 7

## Data Availability

The raw data generated and analyzed during the current study are available from the corresponding author on reasonable request.

## List of abbreviations

ASC: Adipose-derived stromal cells
CFSE: Carboxyfluorescein succinimidyl ester
CIK: Cytokine-induced killer
CPD: Cumulative population doubling
DC: Dendritic cell
FBS: Fetal bovine serum
FCS: Fetal calf serum
GMP: Good Manufacturing Practice
hPL: Human platelet lysate
HS: Human serum
PBMC: Peripheral blood mononuclear cell
PD: Population doubling
PI: Propidium iodide
UCB: Umbilical cord blood
