# Supplementary figures and images for: The effect of serum origin on cytokines induced killer cell expansion and function
Source: BMC Immunol. 2023 Sep 1;24:28. doi: 10.1186/s12865-023-00562-3 (PMC10474620; doi:10.1186/s12865-023-00562-3)

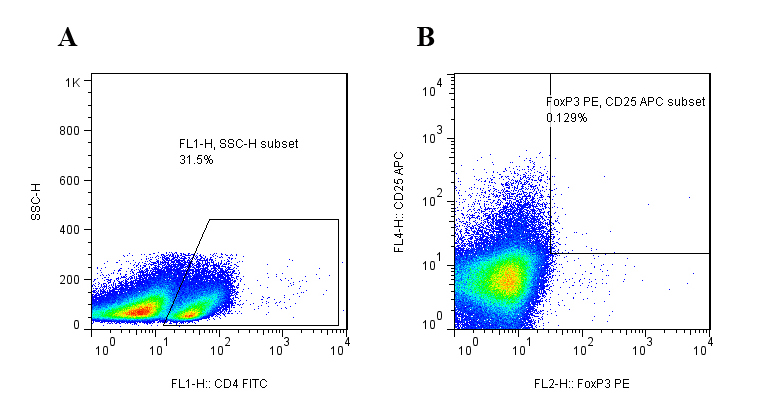

Supplement: Supplementary file 1 — Supplementary Material 1 [file 12865_2023_562_MOESM1_ESM.jpg]
