# Supplementary material for: The effect of serum origin on cytokines induced killer cell expansion and function
Source: BMC Immunol. 2023 Sep 1;24:28. doi: 10.1186/s12865-023-00562-3 (PMC10474620; doi:10.1186/s12865-023-00562-3)

A. Co-culture K562 (Labeled with CFSE) (50,000) + CIK (Cultured in PL 10%) (500,000)


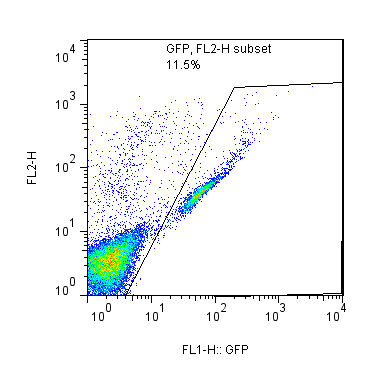

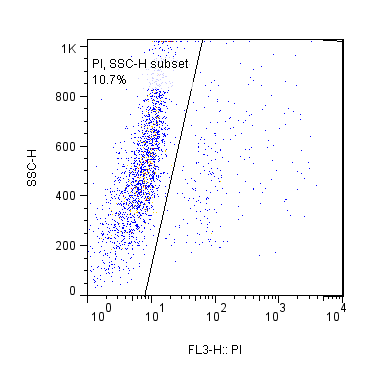


B.Co-culture K562 (Labeled with CFSE) (50,000) + CIK (Cultured in PL 10%) (1,000,000)


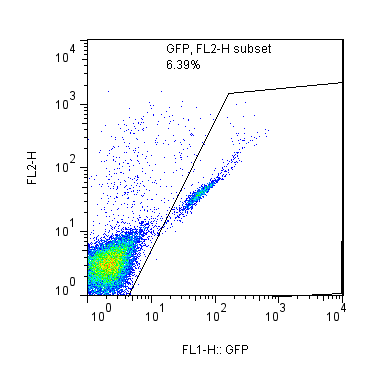

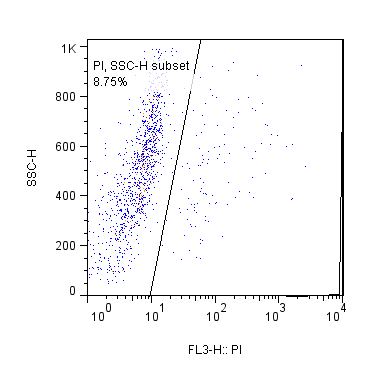


C.Co-culture K562 (Labeled with CFSE) (50,000) + CIK (Cultured in PL 10%) (2,000,000)


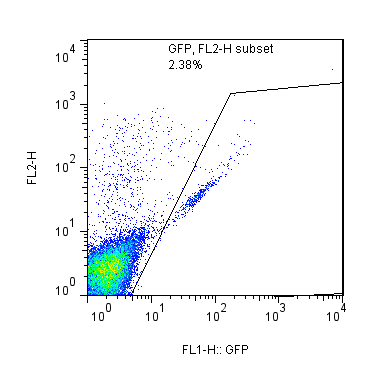

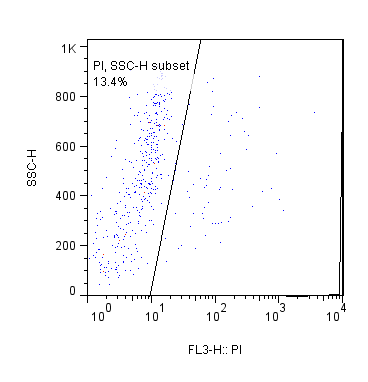


D.Co-culture K562 (Labeled with CFSE) (50,000) + CIK (Cultured in PL 5%) (500,000)


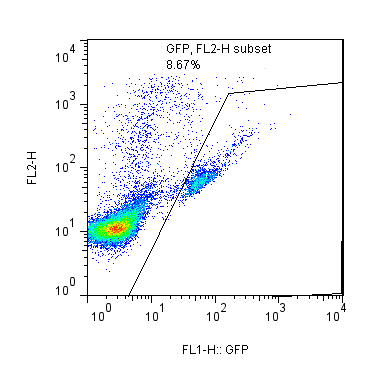

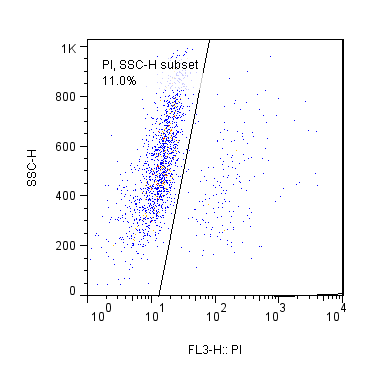


E.Co-culture K562 (Labeled with CFSE) (50,000) + CIK (Cultured in PL 5%) (1,000,000)


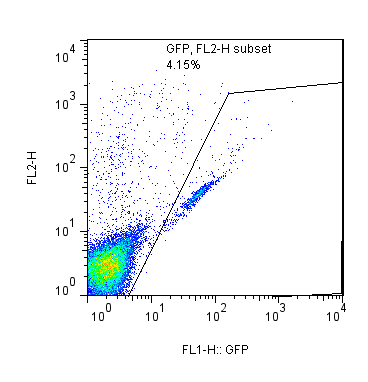

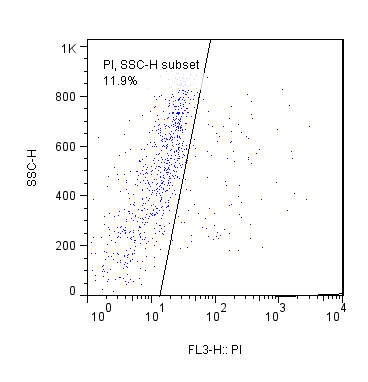


F.Co-culture K562 (Labeled with CFSE) (50,000) + CIK (Cultured in PL 5%) (2,000,000)


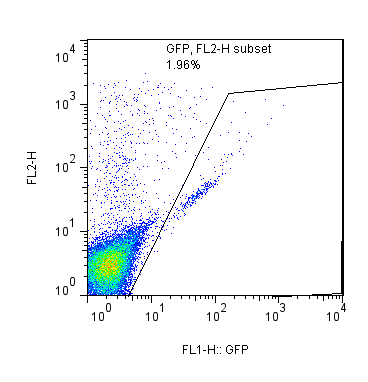

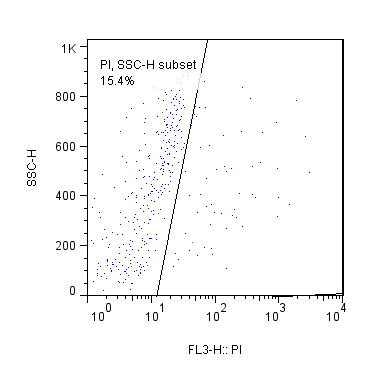


G.Co-culture Raji (Labeled with CFSE) (50,000) + CIK (Cultured in PL 10%) (500,000)


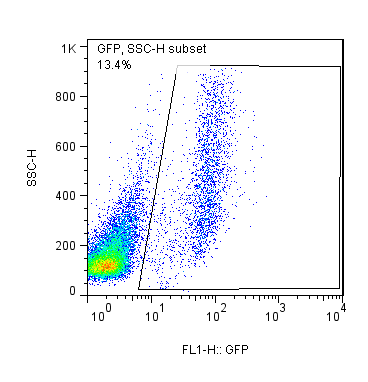

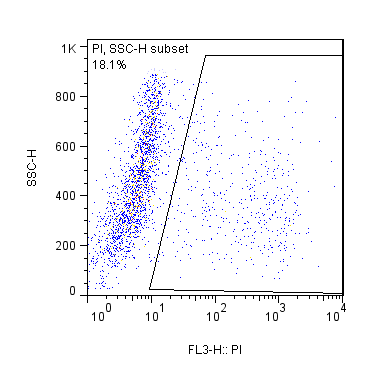


H.Co-culture Raji (Labeled with CFSE) (50,000) + CIK (Cultured in PL 10%) (1,000,000)


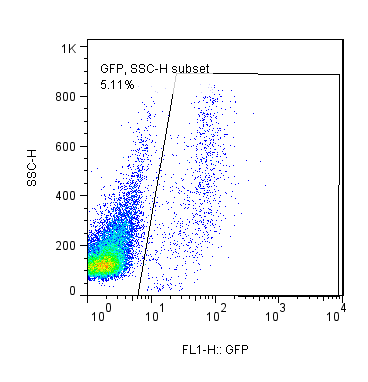

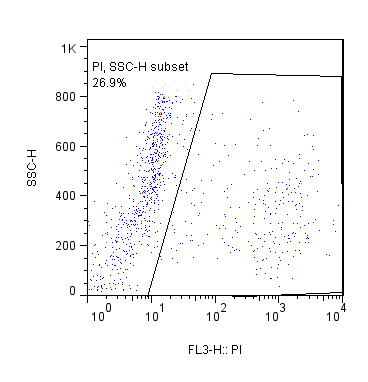


I.Co-culture Raji (Labeled with CFSE) (50,000) + CIK (Cultured in PL 10%) (2,000,000)


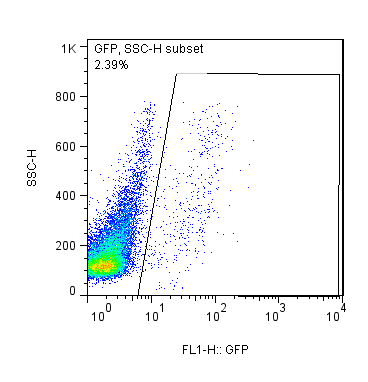

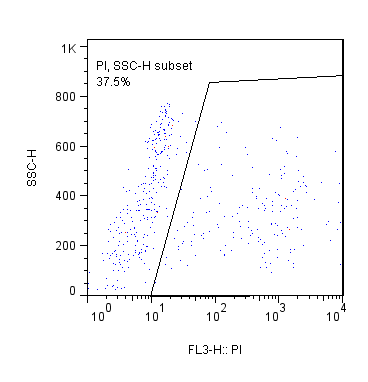


J.Co-culture Raji (Labeled with CFSE) (50,000) + CIK (Cultured in PL 5%) (500,000)


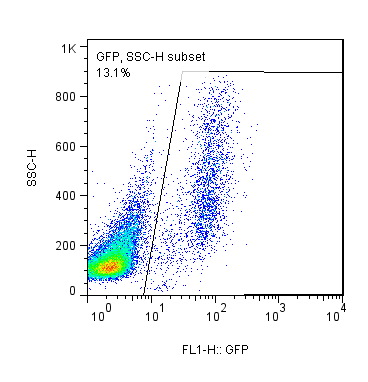

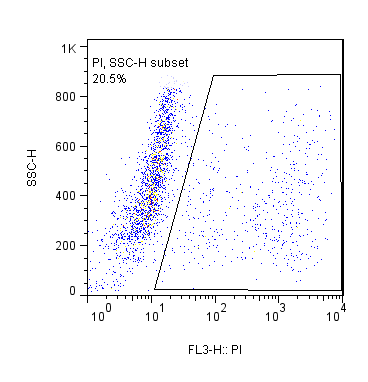


K.Co-culture Raji (Labeled with CFSE) (50,000) + CIK (Cultured in PL 5%) (1,000,000)


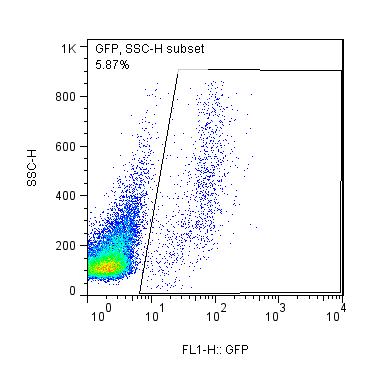

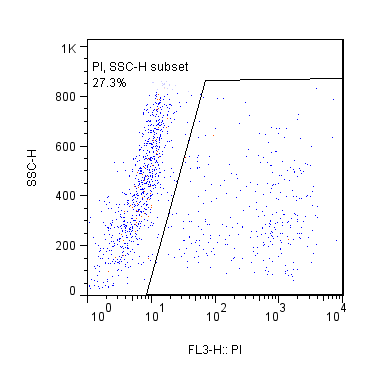


L.Co-culture Raji (Labeled with CFSE) (50,000) + CIK (Cultured in PL 5%) (2,000,000)


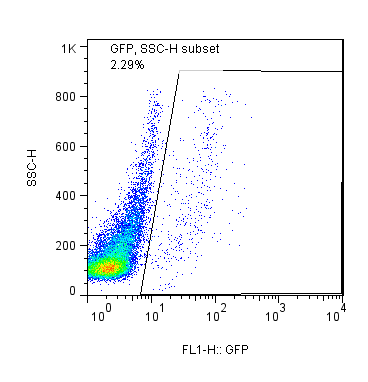

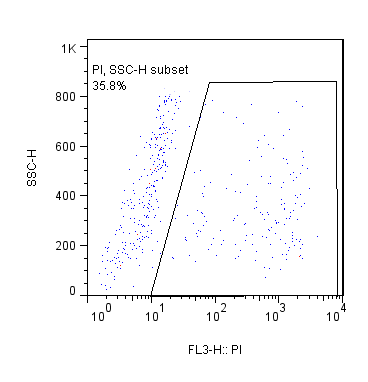

Supplement: Supplementary file 2 — Supplementary Material 2 [file 12865_2023_562_MOESM2_ESM.docx]
