# Supplementary material for: The effect of serum origin on cytokines induced killer cell expansion and function
Source: BMC Immunol. 2023 Sep 1;24:28. doi: 10.1186/s12865-023-00562-3 (PMC10474620; doi:10.1186/s12865-023-00562-3)
Supplement: Supplementary file 3 — Supplementary Material 3 [file 12865_2023_562_MOESM3_ESM.docx]

| **PBMC/ day 0** | | |
| --- | --- | --- |
| CD 3 | CD 3/56 | CD 56 |
| 65 | 16.5 | 1 |

| **hPL 10%** | | | **hPL 5%** | | | **hPL 2.5%** | | |
| --- | --- | --- | --- | --- | --- | --- | --- | --- |
| CD 3 | CD 3/56 | CD 56 | CD 3 | CD 3/56 | CD 56 | CD 3 | CD 3/56 | CD 56 |
| 98.2 | 56.2 | 1.05 | 98.7 | 42.8 | 1.1 | 96.3 | 31.2 | 1.75 |
|  |  |  |  |  |  |  |  |  |
|  |  |  |  |  |  |  |  |  |
| **FBS 10%** | | | **FBS 5%** | | | **FBS 2.5%** | | |
| CD 3 | CD 3/56 | CD 56 | CD 3 | CD 3/56 | CD 56 | CD 3 | CD 3/56 | CD 56 |
| 98.9 | 54.7 | 0.773 | 96.2 | 28.8 | 2.91 | 97 | 21 | 2.38 |
|  |  |  |  |  |  |  |  |  |
|  |  |  |  |  |  |  |  |  |
| **HS 10%** | | | **HS 5%** | | | **HS 2.5%** | | |
| CD 3 | CD 3/56 | CD 56 | CD 3 | CD 3/56 | CD 56 | CD 3 | CD 3/56 | CD 56 |
| 98.5 | 42.4 | 0.876 | 95.4 | 24.4 | 2.33 | 99.2 | 20.9 | 0.65 |
