# Supplementary material for: The effect of serum origin on cytokines induced killer cell expansion and function
Source: BMC Immunol. 2023 Sep 1;24:28. doi: 10.1186/s12865-023-00562-3 (PMC10474620; doi:10.1186/s12865-023-00562-3)
Supplement: Supplementary file 4 — Supplementary Material 4 [file 12865_2023_562_MOESM4_ESM.docx]

| **Fold Expansion (TP 10)** | | | | | | | | |  | **PD/Day (TP 10)** | | | | | | | | |  | **CPD (TP 10)** | | | | | | | | |
| --- | --- | --- | --- | --- | --- | --- | --- | --- | --- | --- | --- | --- | --- | --- | --- | --- | --- | --- | --- | --- | --- | --- | --- | --- | --- | --- | --- | --- |
| hPL 2.5% | | | hPL 5% | | | hPL 10% | | |  | hPL 2.5% | | | hPL 5% | | | hPL 10% | | |  | hPL 2.5% | | | hPL 5% | | | hPL 10% | | |
| Mean | SEM | N | Mean | SEM | N | Mean | SEM | N |  | Mean | SEM | N | Mean | SEM | N | Mean | SEM | N |  | Mean | SEM | N | Mean | SEM | N | Mean | SEM | N |
| 74.44 | 13.03 | 3 | 585.45 | 250.28 | 3 | 3020.86 | 1205.41 | 3 |  | 0.03 | 0.05 | 3 | 0.23 | 0.07 | 3 | 0.25 | 0.04 | 3 |  | 6.53 | 0.40 | 3 | 9.22 | 0.82 | 3 | 11.10 | 0.73 | 3 |
| HS 2.5% | | | HS 5% | | | HS 10% | | |  | HS 2.5% | | | HS 5% | | | HS 10% | | |  | HS 2.5% | | | HS 5% | | | HS 10% | | |
| Mean | SEM | N | Mean | SEM | N | Mean | SEM | N |  | Mean | SEM | N | Mean | SEM | N | Mean | SEM | N |  | Mean | SEM | N | Mean | SEM | N | Mean | SEM | N |
| 72.19 | 71.11 | 3 | 79.13 | 77.023 | 3 | 807.63 | 361.47 | 3 |  | -0.1 | 0.13 | 3 | 0.04 | 0.06 | 3 | 0.10 | 0.05 | 3 |  | 2.63 | 2.57 | 3 | 3.35 | 2.27 | 3 | 8.77 | 0.94 | 3 |
| FBS 2.5% | | | FBS 5% | | | FBS 10% | | |  | FBS 2.5% | | | FBS 5% | | | FBS 10% | | |  | FBS 2.5% | | | FBS 5% | | | FBS 10% | | |
| Mean | SEM | N | Mean | SEM | N | Mean | SEM | N |  | Mean | SEM | N | Mean | SEM | N | Mean | SEM | N |  | Mean | SEM | N | Mean | SEM | N | Mean | SEM | N |
| 1.11 | 0.08 | 3 | 9.35 | 6.94 | 3 | 284.01 | 107.32 | 3 |  | -0.11 | 0.08 | 3 | -0.08 | 0.07 | 3 | 0.10 | 0.01 | 3 |  | 0.16 | 0.11 | 3 | 1.20 | 0.02 | 3 | 7.03 | 0.59 | 3 |
