# Supplementary material for: The effect of serum origin on cytokines induced killer cell expansion and function
Source: BMC Immunol. 2023 Sep 1;24:28. doi: 10.1186/s12865-023-00562-3 (PMC10474620; doi:10.1186/s12865-023-00562-3)
Supplement: Supplementary file 6 — Supplementary Material 6 [file 12865_2023_562_MOESM6_ESM.docx]

| **Viability** | | | | | | | | |  | **Viability** | | | | | | | | |  |
| --- | --- | --- | --- | --- | --- | --- | --- | --- | --- | --- | --- | --- | --- | --- | --- | --- | --- | --- | --- |
| FBS 2.5% | | | hPL 2.5% | | | HS 2.5% | | |  | hPL 2.5% | | | hPL 5% | | | hPL 10% | | |  |
| Mean | SEM | N | Mean | SEM | N | Mean | SEM | N |  | Mean | SEM | N | Mean | SEM | N | Mean | SEM | N |  |
| 61.60 | 0.84 | 3 | 94.67 | 0.88 | 3 | 75.78 | 1.30 | 3 |  | 94.67 | 0.88 | 3 | 95 | 0.58 | 3 | 95.1 | 0.51 | 3 |  |
| FBS 5% | | | hPL 5% | | | HS 5% | | |  | HS 2.5% | | | HS 5% | | | HS 10% | | |  |
| Mean | SEM | N | Mean | SEM | N | Mean | SEM | N |  | Mean | SEM | N | Mean | SEM | N | Mean | SEM | N |  |
| 72.82 | 0.80 | 3 | 95 | 0.58 | 3 | 85.90 | 1.19 | 3 |  | 75.8 | 1.30 | 3 | 85.90 | 1.19 | 3 | 91.77 | 0.74 | 3 |  |
| FBS 10% | | | hPL 10% | | | HS 10% | | |  | FBS 2.5% | | | FBS 5% | | | FBS 10% | | |  |
| Mean | SEM | N | Mean | SEM | N | Mean | SEM | N |  | Mean | SEM | N | Mean | SEM | N | Mean | SEM | N |  |
| 89.94 | 3.35 | 3 | 95.1 | 0.51 | 3 | 91.77 | 0.74 | 3 |  | 61.60 | 0.84 | 3 | 72.82 | 0.80 | 3 | 89.9 | 3.35 | 3 |  |
|  |  |  |  |  |  |  |  |  |  |  |  |  |  |  |  |  |  |  |  |
