# Supplementary material for: The effect of serum origin on cytokines induced killer cell expansion and function
Source: BMC Immunol. 2023 Sep 1;24:28. doi: 10.1186/s12865-023-00562-3 (PMC10474620; doi:10.1186/s12865-023-00562-3)
Supplement: Supplementary file 7 — Supplementary Material 7 [file 12865_2023_562_MOESM7_ESM.docx]

**Supplementary data legends**

**Figure S1. The percentage of Treg cells**

The percentage of Treg cells in CIK cells which is cultured in hPL at TP 10 was evaluated by flow cytometry.

**Figure S2. Cytotoxicity assay (Flow cytometry** **data)**

Co-culture of CFSE-labeled target cells (K562 and Raji) and effector cells (CIK) which is cultured in different concentrations of PL at three effectors to target (E:T) ratios

**Tables**

**Table S1**

The surface expression of CIK CD markers (CD3/CD56) after isolation and culture in different groups (days 0 and 15^th^).

**Table S2**

SEM and Mean of Figure 3 is shown in Tables S1.

**Table S3**

SEM and Mean of Figure 4 is shown in Tables S2.

**Table S4**

SEM and Mean of Figure 5 is shown in Tables S3.
